# Development of heart-sparing VMAT radiotherapy technique incorporating heart substructures for advanced NSCLC patients

**DOI:** 10.1186/s13014-025-02597-9

**Published:** 2025-03-14

**Authors:** Linda Agolli, Ann-Katrin Exeli, Uwe Schneider, Sandra Michaela Ihne-Schubert, Andreas Lurtz, Daniel Habermehl

**Affiliations:** 1 Department of Radiation Oncology, Justus-Liebig-University Giessen, Giessen-Marburg University Hospital, Giessen, Klinikstraße Germany; 2 Department of Internal Medicine IV, University Hospital Gießen and Marburg, Giessen, Germany; 3 Department of Internal Medicine II, University Hospital Würzburg, Würzburg, Germany; 4 CIRCLE - Centre of Innovation Research, Lund University, Lund, Sweden

**Keywords:** Heart substructures, Central non-small cell lung cancer, Active heart sparing, Definitive radiotherapy

## Abstract

**Objective:**

To investigate the feasibility of active heart sparing (AHS) planning in patients with locally advanced and centrally located NSCLC receiving standard definitive radiotherapy (RT), while maintaining or improving appropriate lung, esophagus, and spinal cord constraints and planning target volume (PTV) coverage intent.

**Methods and materials:**

A total of 27 patients with stage IIIA/B NSCLC treated with curative intent RT were selected for this analysis. All existing radiation plans were revised and 27 further new equivalent plans were calculated using AHS for the same cohort of patients. Primary end-point was feasibility of AHS using constraints for heart substructures. The secondary end point was to calculate the difference in terms of dosimetric parameters of heart substructures and principal OARs as well as PTV-coverage parameters within the current patient group.

**Results:**

AHS was feasible in the entire group of patients. An optimal coverage of the target volume was obtained and all mandatory constraints for OARs have been met. The median value of the mean heart dose (MHD) was 8.18 Gy and 6.71 Gy in the standard planning group and AHS-group, respectively (*p* = 0.000). Other heart parameters such as V_5Gy_ (40.57% vs. 27.7%; *p* = 0.000) and V_30Gy_ (5.39% vs. 3.86%; *p* = 0.000) were significantly worse in the standard planning group. The following relevant dosimetric parameters regarding heart substructures were found to be significantly worse in the standard planning group compared to the AHS-group: median dose to heart base (16.97 Gy vs. 6.37 Gy, *p* = 0.000), maximum dose (18.64 Gy vs. 6.05 Gy, *p* = 0.000) and V_15Gy_ (11.11% vs. 0% *p* = 0.000) to LAD; mean dose; V_5Gy_ (9.55% vs. 0.94%, *p* = 0.000) and V_23Gy_ (0.00% vs. 0.00% maximum 45.68% vs. 6.57%, *p* = 0.002 to the left ventricle.

**Conclusion:**

Our analysis showed an improvement of dosimetric parameters of the heart and heart substructures in patients affected by locally advanced and centrally located NSCLC treated with curative RT using AHS optimization. This approach could lead to a possible reduction of heart events and a prolonged survival. New clinical studies regarding RT in advanced NSCLC should include cardiologic evaluations and biomarkers as well as the contouring of cardiac substructures.

**Supplementary Information:**

The online version contains supplementary material available at 10.1186/s13014-025-02597-9.

## Introduction

Cardiac radiation exposure has been identified to be predictive of survival and major cardiac events in patients with locally advanced non-small cell lung cancer (LA-NSCLC) after thoracic radiotherapy (RT) [1–2]. About 35% of patients with are diagnosed in advanced non-metastatic stage and their 2-year overall survival (OS) ranges from 25 to 55% [3]. These patients often present with very large primary central tumors and/or advanced locoregional lymph node metastases. RT is a mainstay in the treatment of LA-NSCLC together with systemic therapy. In the setting of definitive RT, the heart and heart substructures as well as lungs and esophagus can be exposed to high radiation doses [4]. Recently, there is an increasing interest in dose exposure of heart substructures such as the left anterior descending coronary artery (LAD) and heart base that has been shown to play a role in increasing the risk of coronary heart disease and other cardiac diseases [2, 5–6].

Accurate dosimetric and risk assessment studies on heart dose in breast cancer patients motivate the evaluation of the dose to the heart substructures to derive new heart dose constraints and the importance of cardiac evaluation as part of the clinical examination ahead of RT start [7–8].

Studies regarding NSCLC patients treated with RT had shown that doses to specific heart substructures can be associated with different types of cardiac events, such as pericarditis, ischemia and arrhythmia, maybe due to different damage mechanisms that involve pericardium, heart muscles, electric conduction system or vascular structures in the small vessels [9–10]. These findings underline the immediate development of planning methods regarding an active heart sparing and contouring of heart substructures to evaluate the dose distribution within the heart [7].

Our previous research in the field of advanced NSCLC patients demonstrated that dosimetric parameters of LAD were significantly worse after adaptive definitive RT because not considered in the further plan optimization as not routinely contoured [11]. In the new technological era, we need elaborated OARs contouring and novel constraints to improve heart dose including an active heart sparing in the plan optimization.

The aim of our study was to investigate the feasibility of active heart sparing planning in patients with LA- and centrally located NSCLC receiving standard definitive RT, while maintaining or improving appropriate lung, esophagus, and spinal cord constraints and planning target volume (PTV) coverage intent. The difference in terms of dosimetric parameters in heart substructures and principal OARs such as lung, heart and esophagus as well as PTV-coverage parameters within the current patient group with or without active heart sparing (AHS) were assessed in both planning methods. To our current knowledge, this is the first analysis reporting detailed dosimetric data including all cardiac substructures using an AHS optimization approach.

## Patients and methods

### Patients´ characteristics

Twenty-seven patients with stage IIIA/B NSCLC treated with curative intent RT with or without chemotherapy were selected for this analysis. All patients were previously staged and had a histological confirmed diagnosis of NSCLC. Irradiated patients having primary tumors and/or involved lymph nodes at heart level defined as OAR heart + 2 cm in craniocaudal direction were classified as central tumors relevant for the purpose of the current study. The patient population consisted of 14 men and 13 women with a median age of 65 years (range: 59–81) with either adenocarcinoma (*n* = 9) or squamous cell carcinoma (*n* = 17) or other (large cell *n* = 1). The primary tumor was located on the left side of thorax in 16 patients (upper lobe *n* = 9, lower lobe *n* = 5, and central/hilus *n* = 2, respectively); or on the right side of thorax in 11 patients (upper lobe *n* = 7, lower lobe *n* = 2, central/hilus *n* = 2, respectively). Involved lymph nodes were situated as follows: right in 6 patients, left in 8 patients and both sides/median in 11 patients; no nodal involvement in 2 patients.

### Treatment planning and active heart sparing

All patients had a planning CT scan with 2 mm slices and a volumetric modulated arc therapy (VMAT) treatment planning. PET-CT imaging was available and co-registration with the planning CT scan was performed for target volume definition. The gross tumor volume (GTV) encompasses the primary tumor and the positive mediastinal lymph nodes. GTVs were expanded to a total of 5 mm for the primary tumor and 0–2 mm for the involved mediastinal nodes in all directions and anatomically adapted to generate clinical target volumes (CTVs). Afterwards, CTVs were expanded 5 mm in all directions to generate planning target volumes (PTVs).

Auto-Planning will be performed in our treatment planning system (TPS) with standardized target and OAR optimization goal. Prescription to the PTV was according to ICRU 83, dose calculation was done with heterogeneity corrections.

Definitive RT dose was 60–66 Gy in daily 2 Gy single doses. A daily CBCT was performed to verify positioning. Commonly used dose constraints for OARs will be taken into account: whole lung V_20Gy_ < 35% (mandatory) and V_5Gy_ < 65% (preferred, but not mandatory), mean lung dose (MLD) < 20 Gy; heart: mean heart dose (MHD) < 20 Gy, V_50Gy_ < 25% [12]; spinal canal + 3 mm (PRV) max dose < 45 Gy; esophagus V_55Gy_ ≤ 33%, V_60Gy_ ≤ 7% [13], maximal dose < 105% of prescribed dose. The optimization of plan was done using the above constraints also for tumors infiltrating or in close contact with esophagus, but target coverage was prioritized.

**Fig. 1:**
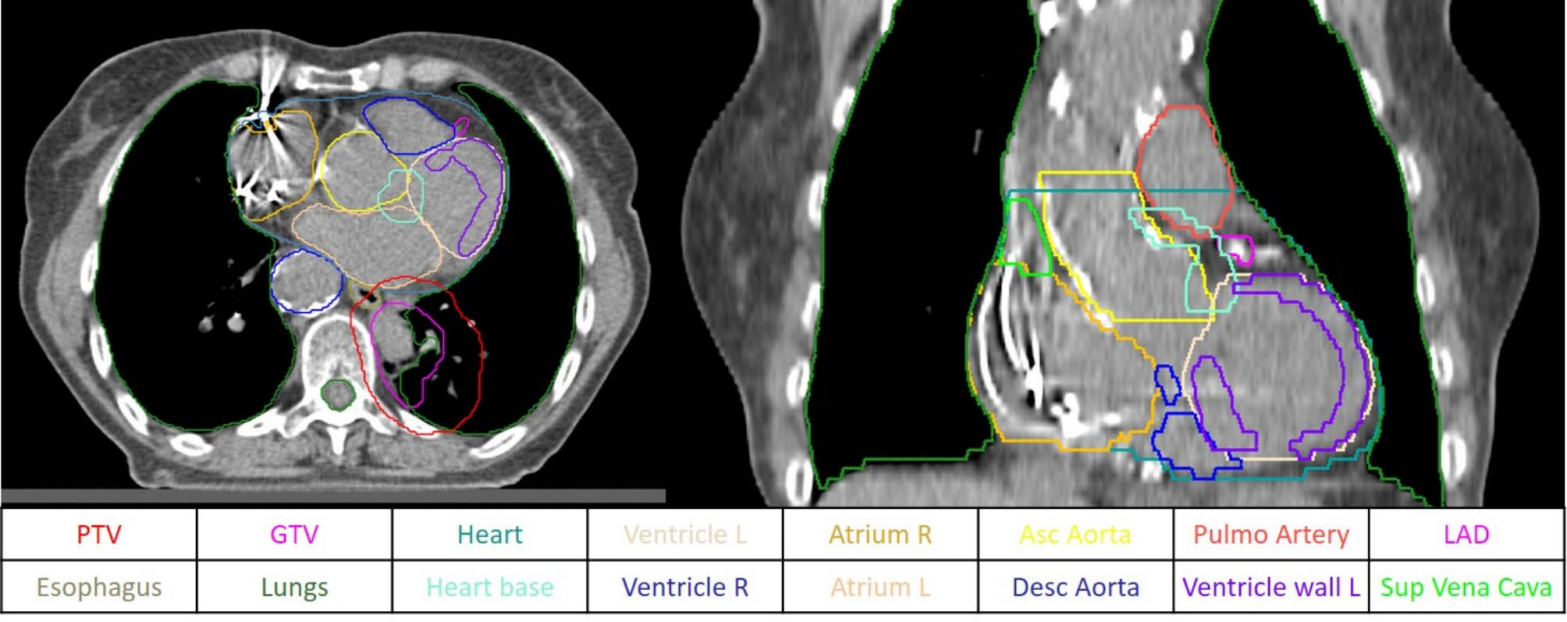
Contouring of organs at risk and cardiac substructures in axial images of planning-CT

Initially, heart substructures were not routinely taken into account for the optimization and the administration of radiation therapy. For the purpose of this study, planning CT scans and related contours from all patients were revised. Cardiac substructures such as atriums, ventricles, LAD and large vessels (ascending/arch, descending aorta, superior vena cava, and pulmonary artery) were retrospectively contoured using an artificial intelligence program (ART-Plan™ TheraPanacea, France) and were then reviewed and approved by an expert radiation oncologist (see Fig. 1), also based on a heart atlas [14].

The base of the heart is a region including the origin of the left coronary artery and the location of the sinoatrial node; this region was found to have a statistic significant impact on survival (*p* = 0.02) [15], and will be contoured manually from an expert radiation oncologist.

**Fig. 2:**
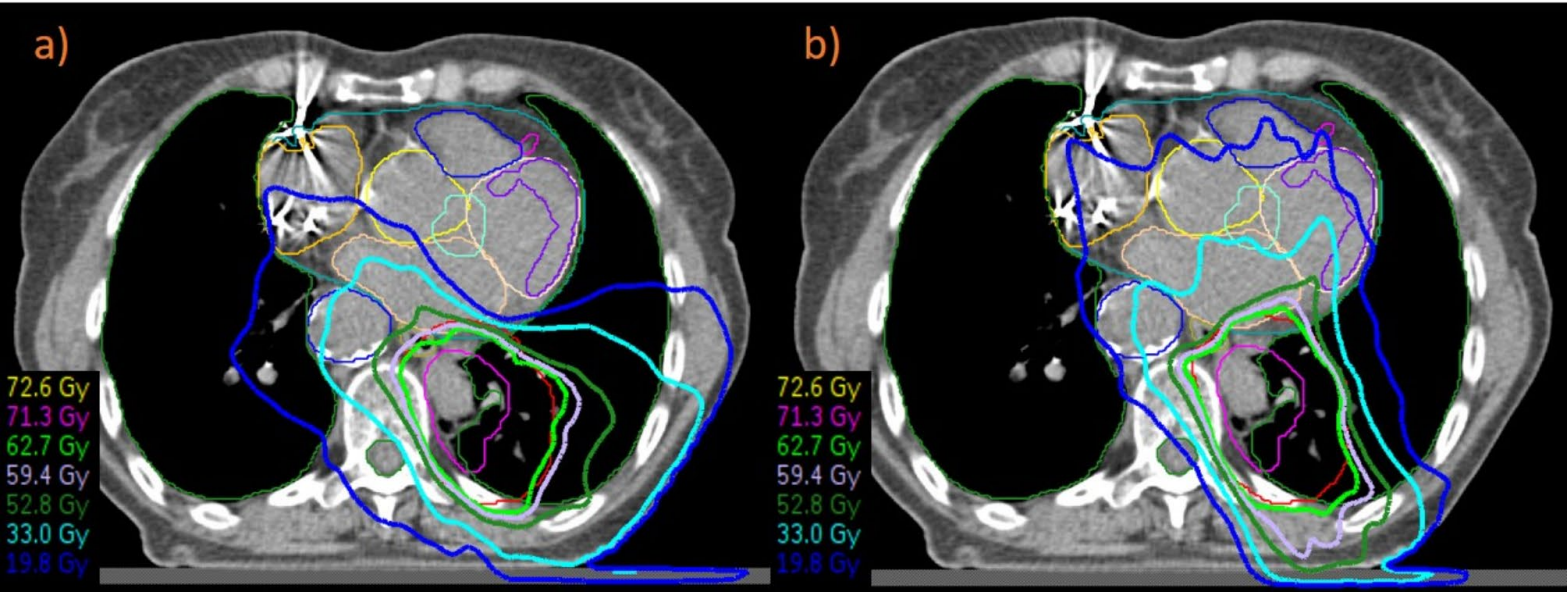
Planning without (left) and with (right) active heart sparing in a patient with central advanced non-small cell lung cancer

Constraints for active heart protection planning were as follows: heart mean < 10 Gy [16], V_30Gy_ < 21% (mandatory) [17]; left ventricle (LV) [18] mean < 3 Gy, V_5Gy_ < 17%, V_23Gy_ < 5%; LAD [5, 18–19] maximal dose 17 Gy, V_15Gy_ < 10%, V_30Gy_ < 2%; heart base (SA-node region) mean < 9 Gy (preferred), mean ≤ 9.1 Gy (mandatory) [15] above (for more details see also Supplement 1/ Table 1 Ref. 3–8); other substructures ALARA. Constraints regarding other OARs were the same as reported above. We generated equivalent plans with AHS, where equal nominal energy beams and target dose homogeneity were employed, along with coverage of at least 95% of the target volume with the same prescribed dose, resulting in an equivalent dose distribution (ICRU 83) (see Fig. 2).

**Table 1:** Mandatory constraints for AHS RT-planning

OAR in active heart sparing	Parameter	
**Heart**	V30 Gy ≤ 21%	mandatory
**LAD**	V15Gy < 10%	mandatory
**Heart base**	D mean ≤ 9.1 Gy	mandatory

### End points and statistical analysis

The objective of this study was to investigate the feasibility of an active heart sparing planning in patients with LA- and centrally located NSCLC receiving standard definitive RT, while maintaining or improving appropriate lung, esophagus, and spinal cord constraints and PTV coverage intents. To assess the feasibility of the AHS technique, a score was made including only the mandatory constraints that were: heart V_30Gy_ ≤ 21% [17], LAD V_15Gy_ < 10% [18–19], and heart base D_mean_ ≤ 9.1 Gy [15] as reported in Table 1. Complete AHS was defined as 3 met constraints; partial AHS was defined as 1–2 met constraints; no AHS was defined as no met constraints.

A further objective of the study was to calculate the difference in terms of dosimetric parameters in heart substructures and principal OARs as well as PTV-coverage parameters within the current patient group with or without active heart sparing planning method.

Dosimetric parameters regarding OARs and newly contoured heart substructures well as GTV/PTV parameters were extracted and descriptive statistics such as mean values, standard deviations, medians, minimum and maximum values were calculated using STATA version 18.5 BE. For the comparison of the dosimetric parameters of original plans group and AHS plans group the differences of each parameter of the original and AHS plan group were calculated. If both planning methods are equivalent, the measure of central tendency (median or mean value) of the difference is zero which was defined as null hypothesis to be tested. Then a one-sample test was used to test whether the median or the mean of the differences of each parameter differs from zero. The one-sample t-test was used for the mean values and the Wilcoxon signed rank test was performed for the medians. A regression analysis was performed to determine the impact of the primary tumor side (left side = 1; right side = 0) and volume of PTV on the difference of the dosimetric parameters between the planning methods (original plan – AHS plan) regarding heart and heart substructures.

## Results

### Plan parameters and feasibility of active heart sparing

AHS was feasible in the entire group of patients. The three mandatory constraints were met in all RT plans. An optimal coverage of the target volume was obtained and all mandatory constraints for OARs have been met. In the entire AHS-group pf plans (*n* = 27), the mean value of D_2%_ and D_98%_ of PTV were 68.39 Gy (SD 1.485) and 56.64 Gy (SD 16.206), respectively. The mean value of MLD, V_20Gy_ and V_5Gy_ to the lungs were 14.55 Gy (SD 3.896), 25.87% (SD 7.318) and 58.5% (SD 14.872) respectively.

An active heart sparing was feasible and the constraints regarding heart and heart substructures have been met. The mean value of MHD was 6.21 Gy (SD 2.884) and the heart V_30Gy_ was 4.31% (SD 3.140) respectively. The mean value of V_15Gy_, V_30Gy_ and Dmax to the LAD was 0.09% (SD 0.469), 0,00% (SD 0.000), 6.66 Gy (SD 3.324), respectively. The mean value for heart base in the AHS-group was 5.77 Gy (SD 2.003). Parameters regarding coverage of the target and constraints in the OARs and heart parameters of the AHS-group compared to the inherent constraints are summarized in Table 2. Results of the descriptive statistics including mean values of dosimetric parameters and standard deviations of both planning groups (standard plan and AHS-plan) and the *p*-values resulting from the one-sample t-test are summarized in Supplement 2/ Table 2.

**Table 2:** Feasibility of active heart sparing planning regarding coverage of the target and constraints in the OARs and heart parameters (t-test results)

Active heart sparing group			
Structure	Constraint	Parameter	Mean (SD)
*Standard constraints*			
***Lungs***	MLD ≤ 20 Gy	MLD (Gy)	14,546 (3.896)
	V20 Gy ≤ 35%	V20 Gy (%)	25,871 (7.318)
	V5 Gy < 65%	V5 Gy (%)	58,500 (14.872)
***Esophagus***	D mean 34 Gy	D mean (Gy)	18,195 (7.486)
	V55 Gy ≤ 33%	V55 Gy (%)	11,803 (10.517)
	V60 Gy ≤ 7%	V60 Gy (%)	6,450 (8.526)
	maximal dose < 105% of prescribed dose	Vol > 105% prescribed dose (cc)	0,004 (0.020)
*Heart sparing constraints*			
***Heart***	MHD ≤ 10 Gy	MHD (Gy)	6,208 (2.884)
	V30 Gy < 20%	V30 Gy (%)	4,305 (3.140)
***Left ventricle***	D mean < 3 Gy	D mean Gy	2,276 (1.756)
	V5 Gy < 17%	V5 Gy (%)	8,061 (17.065)
	V23 Gy < 5%	V23 Gy (%)	0,267 (1.262)
***LAD***	D max < 17 Gy	D max Gy	6,658 (3.324)
	V15Gy < 10%	V15 Gy (%)	0,090 (0.469)
	V30 Gy < 2%	V30 Gy (%)	0,000 (0.000)
***Heart base***	D mean < 9 Gy	D mean (Gy)	5,771 (2.003)
**PTV**	Volume (cc)		293,041 (189.244)
	D2% (Gy)		68,388 (1.485)
	D98% (Gy)		56,642 (16.206)

PTV: planning target volume; SD, standard deviation, LAD: left anterior discending coronary artery; D mean: Mean dose; D max: maximal dose; MLD: mean lung dose; MHD: mean heart dose

### Comparison standard planning vs. active heart sparing

The median value of MHD was 8.18 Gy and 6.71 Gy in standard planning group and AHS-group, respectively (*p* = 0.000). Other heart parameters such as V_5Gy_ (40,57% vs. 27,7%; *p* = 0.000) and V_30Gy_ (5.39% vs. 3.86%; *p* = 0.000) were significantly worse in the standard planning group. The following relevant dosimetric parameters regarding heart substructures were found to be significantly worse in the standard planning group compared to the AHS-group: median dose to heart base (16.97 Gy vs. 6.37 Gy, *p* = 0.000), maximum dose (18.64 Gy vs. 6.05 Gy, *p* = 0.000) and V_15Gy_ (11.11% vs. 0%, *p* = 0.000) to LAD; mean dose, V_5Gy_ (9.55% vs. 0.94%, *p* = 0.000) and V_23Gy_ (0.00% vs. 0.00% maximum 45.68% vs. 6.57%, *p* = 0.000) to the left ventricle.

Within the AHS-group the following parameters were significantly higher compared to original plans: V_20Gy_ both lungs (21.68% vs. 25.53%, *p* = 0.000), V_30Gy_ (11.64% vs. 16.05%, *p* = 0.032) both lungs, and V_55Gy_ to esophagus (7.57% vs. 12.39%, *p* = 0.000), even though the lung constraints were always met. Other dosimetric paramters regarding lung and esophagus were non significantly different in both planning groups.

Parameters regarding coverage of the target and constraints in the OARs and heart parameters of the AHS-group compared to standard planning group dosimetric parameters are summarized in Table 3. Results of the descriptive statistics (medians, minimum and maximum values of dosimetric parameters and the *p*-values resulting from the Wilcoxon singed rank test can be found in Supplement 3/ Table 3.

**Table 3:** Comparison of regarding target volume and organs at risk including heart substructures between original plans (*n* = 27) and active heart sparing plans (*n* = 27) in the same patient cohort

Structure	Parameter	Original plan			Heart sparing plan			*p* value
		Median	Minimum	Maximum	Median	Minimum	Maximum	
**PTV**	Volume (cc)	252,504	21,016	867,275	252,504	20,936	867,274	0.2558
	D2 (%)	68,951	67,595	70,161	68,720	61,986	70,154	**0.0385**
	D98 (%)	61,879	8,959	64,691	62,730	8,146	64,382	0.2584
**Heart**	D mean (Gy)	8,177	1,733	21,466	6,707	1,542	11,554	**0.0000**
	V5 Gy (%)	40,565	5,346	94,596	27,704	1,474	71,341	**0.0000**
	V30 Gy (%)	5,390	0,000	23,840	3,861	0,000	11,514	**0.0000**
	V35 Gy (%)	3,883	0,000	20,869	3,369	0,000	10,663	**0.0000**
	V50 Gy (%)	1,671	0,000	10,529	1,438	0,000	8,119	**0.0037**
**Heart base**	D max (Gy)	38,205	11,249	69,306	16,352	4,592	66,772	**0.0000**
	D mean (Gy)	16,966	2,921	32,424	6,355	2,481	10,267	**0.0000**
	D max 1 cc (Gy)	27,650	9,325	60,622	8,870	4,080	42,444	**0.0000**
**Ascending aorta**	D max 0.03 cc (Gy)	41,659	20,889	68,149	28,789	6,839	69,559	**0.0000**
	D max 1 cc (Gy)	37,054	17,807	67,022	23,872	5,581	66,802	**0.0000**
	D mean (Gy)	15,781	5,799	42,634	9,875	2,523	29,158	**0.0000**
**Discending aorta**	D max 0.03 cc (Gy)	68,891	30,383	70,859	67,719	43,129	71,249	0.2584
	D max 1 cc (Gy)	67,250	25,720	70,155	66,831	37,909	69,959	0.4846
	D mean (Gy)	22,238	4,969	46,705	23,929	6,618	43,641	0.0692
**LAD**	D max (Gy)	18,642	4,113	56,238	6,054	1,822	16,494	**0.0000**
	D max 1 cc (Gy)	9,772	1,650	47,053	3,699	1,113	8,023	**0.0000**
	D mean (Gy)	7,794	1,201	34,257	3,031	0,942	6,264	**0.0000**
	V30 Gy (%)	0,000	0,000	63,095	0,000	0,000	0,000	**0.0078**
	V15 Gy (%)	11,111	0,000	91,667	0,000	0,000	2,439	**0.0000**
**Pulmonary artery**	D max 0.03 cc (Gy)	68,899	65,433	71,379	68,379	61,224	71,319	0.1399
	D max 1 cc (Gy)	67,466	46,200	69,551	67,288	40,990	69,846	0.0552
	D mean (Gy)	35,428	16,134	51,099	25,535	8,348	42,331	**0.0000**
**Superior vena cava**	D max 0.03 cc (Gy)	42,888	8,769	68,379	32,053	4,279	69,370	0.5460
	D max 1 cc (Gy)	29,661	6,860	66,956	27,100	2,761	68,216	0.4270
	D mean (Gy)	26,040	5,390	65,847	22,859	2,488	66,409	**0.0410**
**Left atrium**	D max 0.03 cc (Gy)	67,339	12,599	70,999	61,578	6,009	69,919	0.0762
	D max 1 cc (Gy)	51,730	10,077	68,550	53,410	5,083	68,409	0.0619
	D mean (Gy)	14,617	2,178	40,115	11,559	2,216	28,905	**0.0013**
**Right atrium**	D max 0.03 cc (Gy)	19,099	1,609	71,119	13,999	1,729	68,979	**0.0123**
	D max 1 cc (Gy)	10,448	1,458	68,305	12,294	1,570	67,736	**0.0229**
	D mean (Gy)	4,020	0,807	33,310	3,841	0,843	27,352	**0.0076**
**Left ventricle**	D max 0.03 cc (Gy)	13,169	2,619	70,509	7,283	1,719	69,899	**0.0001**
	D max 1 cc (Gy)	9,530	2,353	68,875	5,632	1,450	66,470	**0.0000**
	D mean (Gy)	2,649	0,846	25,066	1,918	0,631	9,143	**0.0000**
	V5Gy (%)	9,547	0,000	99,984	0,938	0,000	72,920	**0.0000**
	V23 Gy (%)	0,000	0,000	45,679	0,000	0,000	6,565	**0.0020**
**Right ventricle**	D max 0.03 cc (Gy)	13,579	1,863	44,259	6,649	1,716	15,319	**0.0001**
	D max 1 cc (Gy)	11,150	1,653	37,038	4,869	1,365	11,463	**0.0000**
	D mean (Gy)	2,575	0,620	13,445	1,530	0,468	4,790	**0.0000**
**Lungs**	MLD (Gy)	13,523	5,067	21,254	13,948	5,108	21,985	**0.0000**
	V5 Gy (%)	61,922	25,759	90,333	56,284	26,216	87,351	0.6790
	V20 Gy (%)	21,675	6,508	36,252	25,529	6,581	38,368	**0.0000**
	V30 Gy (%)	11,639	2,542	28,380	16,046	2,441	27,239	**0.0000**
**Esophagus**	D mean (Gy)	17,951	2,713	33,283	18,908	4,226	30,202	0.0121
	D max (Gy)	66,327	23,511	70,914	65,996	25,809	70,061	0.8408
	V55 Gy (%)	7,570	0,000	41,040	12,386	0,000	35,282	**0.0317**
	V60 Gy (%)	3,314	0,000	35,841	3,479	0,000	31,211	0.2063
	Volume included in 105% isodose (cc) of prescribed dose	0,000	0,000	0,277	0,000	0,000	0,103	0.1250

PTV: planning target volume; GTV: gross tumor volume, SD, standard deviation, LAD: left anterior discending coronary artery; D mean: Mean dose; D max: maximal dose; MLD: mean lung dose; cc: cubic centimeter

### Impact of other factors on dosimetric parameters

**Fig. 3:**
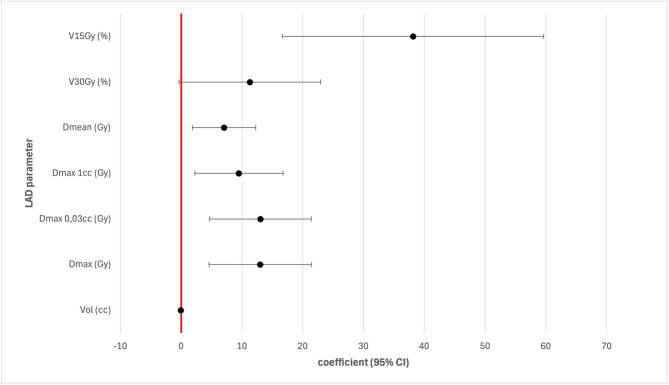
Regression coefficients with the 95% CIs as forest plot for the LAD parameters positively correlating with the left side of the primary tumor

The volume of PTV was found to be mostly unassociated with the dosimetric parameters of heart substructures. However, the laterality of the primary tumor had a significant impact on some dosimetric parameters. Patients with primary tumor located on the left side showed a higher benefit regarding D_max_ (*p* = 0.004), D_0.03cc_ (*p* = 0.004), D_1cc_ (*p* = 0.013), D_mean_ (*p* = 0.010), V_15Gy_ (*p* = 0.001) of the LAD (see Fig. 3). Patients with right-sided tumors showed more pronounced effects regarding Dmax_1cc_ in the left atrium (*p* = 0.027), D_mean_ in the right atrium (*p* = 0.041) and D_mean_ to the superior vena cava (*p* = 0.042).

## Discussion

The proximity of the tumor to the heart, especially in LA-NSCLC, increases the risk of cardiac complications, which can negatively impact overall survival and quality of life. Advanced technology and the use of IMRT or VMAT planning in advanced NSCLC have contributed to safely deliver radiotherapy and to reduce rates of severe lung toxicity and the mean heart dose could be reduced [20–21]. However, the optimal radiotherapy VMAT plans, that achieved high conformity and homogeneity to PTV and minimize the dose to OARs patients with centrally located NSCLC are still not routinely implemented. Principally, heart substructures are not even taken into account. Recent attempts of plan VMAT optimization in this setting showed favorable DVH-parameters in the principal OAR as lung, esophagus and heart [22], but no huge efforts in heart substructures have been done. In 2022, McKenzie et al. reported that LAD V_15Gy_ ≥ 10% was associated with a significant increased risk of all-cause mortality and 2-year OS was significantly lower in patients with LA-NSCLC underwent thoracic RT after a re-analysis of RTOG 0617 series [19].

In our dosimetric study, we aimed to investigate the feasibility of an active heart sparing planning in patients with LA- and centrally located NSCLC receiving standard definitive RT, while maintaining or improving appropriate lung, esophagus, and spinal cord constraints and PTV coverage intent. For this reason, we used specific constraints for the heart and cardiac (see Supplement 1) and standard PTV coverage and OAR constraints. Planning of definitive dose radiation to advanced NSCLC patients using an AHS was feasible in the entire group of patients. An optimal coverage of the target volume was achieved. Other constraints to OARs were not compromised, in particular the mean value of MLD, V_20Gy_ and V_5Gy_ to the lungs were 14.55 Gy, 25.87%, and 58.5%, respectively.

Heart dosimetric parameters such as percent of heart volume receiving ≥ 5 Gy and ≥ 30 Gy are important predictors for survival [23] and should be considered in the optimization for radiation plans. Moreover, Dess et al. reported, that 2-years incidence of grade ≥ 3 cardiac events primarily consisted in acute coronary syndrome exceeded 10% among patients with LA-NSCLC treated with definitive thoracic RT; pre-existing heart disease and higher mean heart dose were significantly associated with higher cardiac event rates [1]. The group advices to reduce heart doses in order to decrease risk of radiation-associated heart injury.

Base on existing literature, we tried to use many constraints in the heart protection planning strategy including the heart and also heart substructures such as LAD, left ventricle and heart base (sinoatrial node region in the heart) [5, 15–18]. The AHS plans were compared with standard plans and improvement of dosimetric parameters was found in the cardiac substructures for the respective constraints but also in other substructures without specific constraints. In addition, the dosimetric parameters of the heart were significantly improved with an AHS optimization by maintaining the necessary parameters for target coverage and other important OARs such as lung and esophagus.

The median value of MHD was 8.18 Gy and 6.71 Gy in standard planning group and AHS-group, respectively (*p* = 0.000). Other heart parameters such as V_5Gy_ (40.57% vs. 27.7%; *p* = 0.000) and V_30Gy_ (5.39% vs. 3.86%; *p* = 0.000) were significantly worse in the standard planning group. Moreover, mean dose to heart base (16.97 Gy vs. 6.37 Gy, *p* = 0.000), maximum dose (18.64 Gy vs. 6.05 Gy, *p* = 0.000) and V_15Gy_ (11.11% vs. 0.000, *p* = 0.000) to LAD; mean dose; V_5Gy_ (9.55% vs. 0.94%, *p* = 0.000) and V_23Gy_ (0.00% vs. 0.00% maximum 45.68% vs. 6.57%, *p* = 0.000) to left ventricle were significantly better in the AHS group compared to standard plans.

The complexity of heart-sparing radiotherapy plans requires a steep learning curve for radiation oncologists, dosimetrists, and medical physicists. Plan optimization in AHS is a time-intensive process due to the complex interplay between tumor control and sparing of OARs. Dose constraints and beam modulations must frequently adjust to find the optimal balance between PTV coverage and heart sparing. In a recent study including 21 academic centers by Herr et al., mean MHD declined from an average of 12.2 Gy to 10.4 Gy (*p* < 0.0001) and the percentage of patients receiving MHD > 20 Gy was reduced from 21.1 to 10.3% (*p* < 0.0001), while MLD and mean esophagus dose did not increase [23]. These achievements were possible by undertaking a years-long process of education and initiation of standardized cardiac dose constraints on heart dose across a statewide consortium.

Efforts are needed to streamline and expedite heart-sparing plan optimization. The integration of artificial intelligence (AI) and machine learning algorithms into treatment planning could help to reduce the time required for plan optimization. AI-driven tools can assist in the automatic delineation.

of OARs and particular heart substructures, preliminary dose optimization, and even generating initial treatment plans based on historical data [24–25]. In addition, standardized heart-sparing protocols for specific clinical scenarios can reduce the time spent on plan optimization using predefined dose constraints and beam configurations tailored to common NSCLC tumor locations.

More and more data are emerging on the survival and major cardiac events benefit in patients already receiving more favorable dosimetric parameters in the heart and cardiac substructures [6, 26]. This leads to the need for prospective studies with use of these parameters as constraints to improve radiotherapy planning in locally advanced lung cancer in clinical practice. Besides planning, a refinement of the contouring of organs at risk by adding cardiac substructures and tumor volumes should be introduced. AI-based programs and standardized treatment plans may help in this process.

## Conclusion

Our analysis showed an improvement of dosimetric parameters in heart substructures, while maintaining optimal OARs constraints and PTV coverage in patients affected by LA- and centrally located NSCLC treated with curative RT. The AHS could lead to a possible reduction of heart events and a prolonged survival. However, a learning time of this planning approach and other resources are required. New research studies, in particular clinical studies regarding RT in advanced NSCLC, should include cardiologic evaluations and biomarkers to determine risk and mechanisms of heart events due to therapy. AHS should be routinely introduced to reduce heart toxicities in the future.

## Electronic supplementary material

Below is the link to the electronic supplementary material.

Supplementary Material 1

Supplementary Material 2

Supplementary Material 3

## Data Availability

No datasets were generated or analysed during the current study.
