# Supplementary material for: Development of heart-sparing VMAT radiotherapy technique incorporating heart substructures for advanced NSCLC patients
Source: Radiat Oncol. 2025 Mar 14;20:40. doi: 10.1186/s13014-025-02597-9 (PMC11908025; doi:10.1186/s13014-025-02597-9)
Supplement: Supplementary file 1 — Supplementary Material 1 [file 13014_2025_2597_MOESM1_ESM.docx]

Table 1. Constraints used in the standard and AHS group up to 66 Gy.

| **Standard planning** | | | **Active heart sparing planning** | | |
| --- | --- | --- | --- | --- | --- |
| **OAR** | **Parameter** |  | **Structure** | **Parameter** |  |
| ***Lungs*** | MLD ≤ 20 Gy |  | ***Heart*** [3-4] | HMD < 10 Gy |  |
|  | V20 Gy ≤ 35% | mandatory |  | V30 Gy < 20% | preferred |
|  | V5 Gy < 65% | preferred |  | V30 Gy ≤ 21% | mandatory |
| ***Heart*** [1] | MHD < 20 Gy |  | ***Left ventricle*** [5] | D mean < 3Gy |  |
|  | V50Gy < 25% |  |  | V5 Gy < 17% |  |
| ***Esophagus*** [2] | D mean 34 Gy |  |  | V23 Gy < 5% |  |
|  | V55 Gy ≤ 33% | preferred | ***LAD*** [5-7] | D max < 17Gy | preferred |
|  | V60 Gy ≤ 7% | preferred |  | V15Gy < 10% | mandatory |
|  | maximal dose < 105% of prescribed dose |  |  | V30 Gy <2% | preferred |
| ***Spinal canal + 3 mm (PRV)*** | D max < 45 Gy |  | ***Heart base*** [8] | D mean < 9 Gy | preferred |
|  |  |  |  | D mean ≤ 9.1 Gy | mandatory |
|  |  |  | ***Other Substructures*** | ALARA |  |

OAR: organ at risk; MLD: mean lung dose; MHD: mean heart dose; LAD: left anterior discending coronary artery: PRV: planning risk volume; ALARA: as low as reasonably achievable

References

1. NCCN Guidelines Version 9.2024 -Non-Small Cell Lung Cancer
2. Palma DA, Senan S, Oberije C, Belderbos J, de Dios NR, Bradley JD, Barriger RB, Moreno-Jiménez M, Kim TH, Ramella S, Everitt S, Rengan R, Marks LB, De Ruyck K, Warner A, Rodrigues G. Predicting esophagitis after chemoradiation therapy for non-small cell lung cancer: an individual patient data meta-analysis. Int J Radiat Oncol Biol Phys. 2013 Nov 15; 87(4):690-6.
3. Wang K, Eblan MJ, Deal AM, Lipner M, Zagar TM, Wang Y, Mavroidis P, Lee CB, Jensen BC, Rosenman JG, Socinski MA, Stinchcombe TE, Marks LB. Cardiac Toxicity After Radiotherapy for Stage III Non-Small-Cell Lung Cancer: Pooled Analysis of Dose-Escalation Trials Delivering 70 to 90 Gy. J Clin Oncol. 2017 May 1;35(13):1387-1394. doi: 10.1200/JCO.2016.70.0229
4. Lehrer EJ, Geyer S, Goodrich A, Ilson DH, Noonan A, Dumane VA, Goodman KA. Impact of Lung and Heart Radiation Dose during Preoperative Chemoradiation on Overall Survival (OS) in Esophageal Cancer (EC) – Secondary Analysis of CALGB 80803 (Alliance). International Journal of Radiation Oncol Biol Phys 2022, Vol 114: 3: S13
5. Whelan TJ, Pignol JP, Levine MN, Julian JA, MacKenzie R, Parpia S, Shelley W, Grimard L, Bowen J, Lukka H, Perera F, Fyles A, Schneider K, Gulavita S, Freeman C. Long-term results of hypofractionated radiation therapy for breast cancer. N Engl J Med. 2010 Feb 11;362(6):513-20.
6. McKenzie E, Zhang S, Zakariaee R, Guthier CV, Hakimian B, Mirhadi A, Kamrava M, Padda SK, Lewis JH, Nikolova A, Mak RH, Atkins KM. Left Anterior Descending Coronary Artery Radiation Dose Association with All-Cause Mortality in NRG Oncology Trial RTOG 0617. Int J Radiat Oncol Biol Phys. 2022 Nov 24:S0360-3016(22)03565-9.).
7. Atkins KM, Chaunzwa TL, Lamba N, Bitterman DS, Rawal B, Bredfeldt J, Williams CL, Kozono DE, Baldini EH, Nohria A, Hoffmann U, Aerts HJWL, Mak RH. Association of Left Anterior Descending Coronary Artery Radiation Dose With Major Adverse Cardiac Events and Mortality in Patients With Non-Small Cell Lung Cancer. JAMA Oncol. 2021 Feb 1;7(2):206-219. doi: 10.1001/jamaoncol.2020.6332.
8. McWilliam A, Abravan A, Banfill A, Faivre-Finn C, van Herk M. Demystifying the Results of RTOG 0617: Identification of Dose Sensitive Cardiac Subregions Associated With Overall Survival. J Thorac Oncol. 2023 May;18(5):599-607. doi: 10.1016/j.jtho.2023.01.085.
