# Supplementary material for: Development of heart-sparing VMAT radiotherapy technique incorporating heart substructures for advanced NSCLC patients
Source: Radiat Oncol. 2025 Mar 14;20:40. doi: 10.1186/s13014-025-02597-9 (PMC11908025; doi:10.1186/s13014-025-02597-9)
Supplement: Supplementary file 2 — Supplementary Material 2 [file 13014_2025_2597_MOESM2_ESM.docx]

Table 2: T-test results of all parameter including target volume, organs at risk and heart substructures (mean, standard deviation and p-value) regarding original plans and active heart sparing plans.

| **Structure** | **Parameter** | **Original plan** | | **Heart sparing plan** | |  |
| --- | --- | --- | --- | --- | --- | --- |
|  |  | ***Mean*** | ***SD*** | ***Mean*** | ***SD*** | ***p value*** |
| **GTV** | Volume (cc) | 137.021 | (126.599) | 136.990 | (126.596) | 0.5044 |
|  | D max (Gy) | 70.494 | (1.034) | 69.770 | (1.947) | **0.0427** |
|  | D mean (Gy) | 64.835 | (5.251) | 64.448 | (5.019) | 0.1367 |
|  | Dose to volume included in 95% isodose (Gy) | 60.110 | (12.377) | 59.755 | (12.643) | 0.4767 |
|  | D1% (Gy) | 69.126 | (0.854) | 68.600 | (1.625) | 0.0962 |
|  | D2% (Gy) | 68.794 | (0.875) | 68.263 | (1.565) | 0.0856 |
|  | D98% (Gy) | 56.906 | (16.310) | 56.877 | (15.813) | 0.9540 |
|  | D99% (Gy) | 56.107 | (17.097) | 56.076 | (16.505) | 0.9481 |
| **PTV** | Volume (cc) | 293.033 | (189.234) | 293.041 | (189.244) | 0.8614 |
|  | D max (Gy) | 71.931 | (0.993) | 70.922 | (2.057) | **0.0027** |
|  | D mean (Gy) | 65.001 | (4.166) | 64.596 | (4.215) | 0.0917 |
|  | Dose to volume included in 95% isodose (Gy) | 57.981 | (15.330) | 58.430 | (14.419) | 0.2866 |
|  | D1% (Gy) | 69.405 | (0.706) | 68.757 | (1.527) | 0.0342 |
|  | D2% (Gy) | 68.998 | (0.698) | 68.388 | (1.485) | 0.0381 |
|  | D98% (Gy) | 56.210 | (16.290) | 56.642 | (16.206) | 0.3238 |
|  | D99% (Gy) | 55.038 | (16.510) | 55.492 | (16.823) | 0.3777 |
| **Heart** | Volume (cc) | 737.612 | (152.105) | 750.060 | (145.377) | 0.3222 |
|  | D max (Gy) | 67.126 | (8.531) | 66.106 | (11.337) | 0.1170 |
|  | D mean (Gy) | 9.928 | (5.788) | 6.208 | (2.884) | **0.0000** |
|  | D max 1 cc (Gy) | 63.368 | (10.211) | 61.849 | (12.925) | **0.0718** |
|  | V5 Gy (%) | 43.355 | (25.296) | 29.828 | (17.952) | **0.0000** |
|  | V30 Gy (%) | 9.059 | (7.794) | 4.305 | (3.140) | **0.0003** |
|  | V35 Gy (%) | 6.956 | (6.418) | 3.569 | (2.777) | **0.0012** |
|  | V50 Gy (%) | 3.231 | (3.369) | 2.109 | (2.003) | **0.0118** |
| **Heart base** | Volume (cc) | 19.142 | (7.129) | 19.199 | (7.110) | 0.0691 |
|  | D max (Gy) | 40.387 | (16.721) | 22.574 | (18.175) | **0.0000** |
|  | D mean (Gy) | 17.017 | (8.933) | 5.771 | (2.003) | **0.0000** |
|  | D max 1 cc (Gy) | 30.339 | (14.480) | 11.123 | (8.230) | **0.0000** |
| **Ascending aorta** | Voume (cc) | 47.724 | (15.786) | 47.718 | (15.786) | 0.6436 |
|  | D max 0.03 cc (Gy) | 44.001 | (14.435) | 34.397 | (16.724) | **0.0000** |
|  | D max 1 cc (Gy) | 38.582 | (14.455) | 28.621 | (15.723) | **0.0000** |
|  | D mean (Gy) | 19.223 | (10.241) | 10.978 | (5.815) | **0.0000** |
| **Discending aorta** | Voume (cc) | 149.939 | (56.443) | 150.301 | (56.149) | 0.7732 |
|  | D max 0.03 cc (Gy) | 62.658 | (12.317) | 62.604 | (9.266) | 0.9615 |
|  | D max 1 cc (Gy) | 58.777 | (14.268) | 59.030 | (11.945) | 0.8523 |
|  | D mean (Gy) | 22.878 | (11.501) | 23.767 | (10.689) | 0.3014 |
| **LAD** | Volume (cc) | 3.123 | (1.104) | 3.153 | (1.123) | 0.2647 |
|  | D max (Gy) | 22.495 | (13.148) | 6.658 | (3.324) | **0.0000** |
|  | D max 0.03cc (Gy) | 21.554 | (12.964) | 6.350 | (3.193) | **0.0000** |
|  | D max 1cc (Gy) | 13.099 | (10.737) | 4.023 | (1.920) | **0.0000** |
|  | D mean (Gy) | 9.997 | (7.792) | 3.180 | (1.419) | **0.0000** |
|  | V30 Gy (%) | 6.744 | (14.127) | 0.000 | (0.000) | **0.0199** |
|  | V15 Gy (%) | 25.982 | (30.221) | 0.090 | (0.469) | **0.0001** |
| **Coronary sinus** | Volume (cc) | 1.280 | (0.811) | 1,340 | (0.782) | 0.2592 |
|  | D max 0.03 cc (Gy) | 4.592 | (6.058) | 4.013 | (4.886) | 0.3393 |
|  | D max 1 cc (Gy) | 1.951 | (2.397) | 1.803 | (2.394) | 0.5816 |
|  | D mean (Gy) | 3.384 | (3.771) | 3.165 | (3.720) | 0.5273 |
| **Left coronary artery** | Volume (cc) | 0.358 | (0.213) | 0.355 | (0.213) | 0.2950 |
|  | D max 0.03 cc (Gy) | 25.968 | (12.027) | 8.431 | (5.585) | **0.0000** |
|  | D max 1 cc (Gy) | 0.000 | (0.000) | 0.000 | (0.000) | N/A |
|  | D mean (Gy) | 21.932 | (10.901) | 6.607 | (2.347) | **0.0000** |
| **Pulmonary artery** | Volume (cc) | 77.940 | (25.540) | 77.965 | (25.570) | 0.4800 |
|  | D max 0.03 cc (Gy) | 68.841 | (1.306) | 68.341 | (2.326) | 0.1746 |
|  | D max 1 cc (Gy) | 66.679 | (4.280) | 65.839 | (5.432) | **0.0259** |
|  | D mean (Gy) | 34.398 | (9.025) | 26.803 | (9.301) | **0.0000** |
| **Superior vena cava** | Volume (cc) | 5.887 | (2.410) | 5.888 | (2.418) | 0.8509 |
|  | D max 0.03 cc (Gy) | 41.072 | (24.170) | 41.283 | (22.722) | 0.8951 |
|  | D max 1 cc (Gy) | 34.652 | (23.295) | 33.121 | (21.071) | 0.3704 |
|  | D mean (Gy) | 29.585 | (21.146) | 26.171 | (18.285) | 0.0474 |
| **Left atrium** | Volume (cc) | 78.877 | (27.632) | 79.244 | (27.857) | 0.2002 |
|  | D max 0.03 cc (Gy) | 58.174 | (15.965) | 56.403 | (16.615) | 0.2130 |
|  | D max 1 cc (Gy) | 51.178 | (18.039) | 48.304 | (20.019) | 0.0697 |
|  | D mean (Gy) | 16.182 | (9.585) | 12.727 | (7.277) | **0.0019** |
| **Right atrium** | Volume (cc) | 75.411 | (21.628) | 77.271 | (20.950) | 0.3149 |
|  | D max 0.03 cc (Gy) | 26.195 | (21.722) | 22.406 | (19.068) | 0.0227 |
|  | D max 1 cc (Gy) | 22.012 | (20.075) | 17.893 | (16.893) | 0.0189 |
|  | D mean (Gy) | 8.397 | (9.467) | 6.098 | (6.157) | 0.0295 |
| **Left ventricle** | Volume (cc) | 188.788 | (42.392) | 192.852 | (35.615) | 0.3222 |
|  | D max 0.03 cc (Gy) | 20.835 | (19.147) | 11.538 | (14.408) | **0.0009** |
|  | D max 1 cc (Gy) | 17.258 | (16.971) | 8.634 | (12.483) | **0.0005** |
|  | D mean (Gy) | 4.867 | (5.638) | 2.276 | (1.756) | **0.0026** |
|  | V5Gy (%) | 23.062 | (29.355) | 8.061 | (17.065) | **0.0002** |
|  | V23 Gy (%) | 4.251 | (10.090) | 0.267 | (1.262) | **0.0310** |
| **Left ventricel wall** | Volume (cc) | 95.135 | (35.066) | 97.333 | (32.933) | 0.3244 |
|  | D max 0.03 cc (Gy) | 20.236 | (19.654) | 12.716 | (17.964) | **0.0009** |
|  | D max 1 cc (Gy) | 16.957 | (18.447) | 9.960 | (16.363) | **0.0004** |
|  | D mean (Gy) | 6.385 | (11.466) | 4.137 | (10.129) | **0.0063** |
| **Right ventricle** | Volume (cc) | 103.719 | (24.794) | 106.144 | (21.111) | 0.3266 |
|  | D max 0.03 cc (Gy) | 16.315 | (12.010) | 6.806 | (3.495) | **0.0001** |
|  | D max 1 cc (Gy) | 13.590 | (10.476) | 5.324 | (2.631) | **0.0001** |
|  | D mean (Gy) | 4.172 | (3.634) | 1.958 | (1.152) | **0.0004** |
| **Lungs** | Volume (cc) | 3653.310 | (1,030.484) | 3675.275 | (1,001.671) | 0.3489 |
|  | MLD (Gy) | 13.265 | (3.849) | 14.546 | (3.896) | **0.0000** |
|  | V5 Gy (%) | 57.669 | (16.920) | 58.500 | (14.872) | 0.6328 |
|  | V20 Gy (%) | 21.575 | (7.359) | 25.871 | (7.318) | **0.0000** |
|  | V30 Gy (%) | 13.225 | (5.551) | 16.273 | (5.997) | **0.0000** |
| **Ipsilateral lung** | Volume (cc) | 1720.590 | (590.267) | 1725.797 | (581.777) | 0.3780 |
|  | D mean (Gy) | 20.329 | (5.719) | 22.176 | (6.452) | **0.0001** |
| **Contralateral lung** | Volume (cc) | 1932.765 | (622.827) | 1950,063 | (612.162) | 0.3244 |
|  | D mean (Gy) | 7.270 | (3.119) | 8.039 | (3.042) | 0.0767 |
| **Esophagus** | Volume (cc) | 37.885 | (9.906) | 38.147 | (9.400) | 0.5848 |
|  | D mean (Gy) | 16.535 | (7.681) | 18.195 | (7.486) | **0.0426** |
|  | D max (Gy) | 59.439 | (14.774) | 61.214 | (11.739) | 0.2306 |
|  | V55 Gy (%) | 9.186 | (10.517) | 11.803 | (10.517) | 0.0582 |
|  | V60 Gy (%) | 6.188 | (9.355) | 6.450 | (8.526) | 0.7343 |
|  | V60 Gy (cc) | 1.849 | (2.416) | 2.103 | (2.575) | 0.3143 |
|  | Volume included in 105% isodose (cc) | 0.028 | (0.072) | 0.004 | (0.020) | 0.1108 |

PTV: planning target volume; GTV: gross tumor volume, SD, standard deviation, LAD: left anterior discending coronary artery; D mean: Mean dose; D max: maximal dose; MLD: mean lung dose; cc: cubic centimeter.
