# Supplementary material for: Development of heart-sparing VMAT radiotherapy technique incorporating heart substructures for advanced NSCLC patients
Source: Radiat Oncol. 2025 Mar 14;20:40. doi: 10.1186/s13014-025-02597-9 (PMC11908025; doi:10.1186/s13014-025-02597-9)
Supplement: Supplementary file 3 — Supplementary Material 3 [file 13014_2025_2597_MOESM3_ESM.docx]

Table 3: Wilcoxon signed-rank test results regarding original plans ((n=27) parameters including target volume, OAR and heart substructures (median, minimum value, maximum value and p-value) compared to active heart sparing plans (n=27).

| **Structure** | **Parameter** | **Original plan** | | | **Heart sparing plan** | | |  |
| --- | --- | --- | --- | --- | --- | --- | --- | --- |
|  |  | ***Median*** | ***Minimum*** | ***Maximum*** | ***Median*** | ***Minimum*** | ***Maximum*** | ***p value*** |
| **GTV** | Volume (cc) | 101.496 | 2.064 | 593.455 | 101.496 | 1.952 | 593.454 | 0.6492 |
|  | D max (Gy) | 70.714 | 68.196 | 72.622 | 69.723 | 63.019 | 73.153 | **0.0362** |
|  | D mean (Gy) | 66.382 | 45.048 | 67.588 | 66.057 | 45.994 | 67.002 | 0.1349 |
|  | Volume included in 95% isodose (cc) | 64.624 | 13.270 | 65.622 | 64.195 | 14.648 | 65.595 | 0.1775 |
|  | D1 (%) | 69.062 | 67.250 | 70.513 | 68.600 | 62.230 | 70.846 | 0.1286 |
|  | D2 (%) | 68.718 | 66.798 | 70.168 | 68.304 | 61.968 | 70.426 | 0.0954 |
|  | D98 (%) | 64.171 | 11.730 | 65.252 | 63.699 | 9.991 | 65.369 | 0.5149 |
|  | D99 (%) | 63.856 | 9.820 | 65.089 | 63.504 | 8.641 | 65.216 | 0.5944 |
| **PTV** | Volume (cc) | 252.504 | 21.016 | 867.275 | 252.504 | 20.936 | 867.274 | 0.2558 |
|  | D max (Gy) | 72.002 | 69.975 | 73.779 | 70.988 | 63.660 | 74.234 | **0.0009** |
|  | D mean (Gy) | 66.096 | 45.639 | 66.962 | 66.044 | 46.166 | 66.379 | 0.0385 |
|  | Volume included in 95% isodose (cc) | 63.285 | 11.404 | 65.104 | 63.545 | 11.104 | 64.726 | 0.3608 |
|  | D1 (%) | 69.430 | 67.929 | 70.601 | 69.051 | 62.285 | 70.729 | **0.0362** |
|  | D2 (%) | 68.951 | 67.595 | 70.161 | 68.720 | 61.986 | 70.154 | **0.0385** |
|  | D98 (%) | 61.879 | 8.959 | 64.691 | 62.730 | 8.146 | 64.382 | 0.2584 |
|  | D99 (%) | 60.797 | 8.258 | 64.332 | 61.864 | 7.288 | 64.161 | 0.3483 |
| **Heart** | Volume (cc) | 727.104 | 497.259 | 1.180.376 | 731.288 | 519.631 | 1.181.748 | 0.0882 |
|  | D max (Gy) | 69.060 | 26.504 | 71.566 | 69.570 | 13.160 | 71.719 | 0.3012 |
|  | D mean (Gy) | 8.177 | 1.733 | 21.466 | 6.707 | 1.542 | 11.554 | **0.0000** |
|  | D max 1 cc (Gy) | 67.138 | 20.387 | 70.139 | 66.379 | 8.724 | 70.026 | 0.1225 |
|  | V5 Gy (%) | 40.565 | 5.346 | 94.596 | 27.704 | 1.474 | 71.341 | **0.0000** |
|  | V30 Gy (%) | 5.390 | 0.000 | 23.840 | 3.861 | 0.000 | 11.514 | **0.0000** |
|  | V35 Gy (%) | 3.883 | 0.000 | 20.869 | 3.369 | 0.000 | 10.663 | **0.0000** |
|  | V50 Gy (%) | 1.671 | 0.000 | 10.529 | 1.438 | 0.000 | 8.119 | **0.0037** |
| **Heart base** | Volume (cc) | 17.136 | 7.552 | 33.393 | 17.320 | 7.712 | 33.392 | 0.9551 |
|  | D max (Gy) | 38.205 | 11.249 | 69.306 | 16.352 | 4.592 | 66.772 | **0.0000** |
|  | D mean (Gy) | 16.966 | 2.921 | 32.424 | 6.355 | 2.481 | 10.267 | **0.0000** |
|  | D max 1 cc (Gy) | 27.650 | 9.325 | 60.622 | 8.870 | 4.080 | 42.444 | **0.0000** |
| **Ascending aorta** | Voume (cc) | 43.984 | 20.928 | 96.408 | 43.984 | 20.928 | 96.305 | 0.8331 |
|  | D max 0.03 cc (Gy) | 41.659 | 20.889 | 68.149 | 28.789 | 6.839 | 69.559 | **0.0000** |
|  | D max 1 cc (Gy) | 37.054 | 17.807 | 67.022 | 23.872 | 5.581 | 66.802 | **0.0000** |
|  | D mean (Gy) | 15.781 | 5.799 | 42.634 | 9.875 | 2.523 | 29.158 | **0.0000** |
| **Discending aorta** | Voume (cc) | 145.557 | 42.872 | 269.595 | 145.557 | 42.872 | 274.599 | 0.6989 |
|  | D max 0.03 cc (Gy) | 68.891 | 30.383 | 70.859 | 67.719 | 43.129 | 71.249 | 0.2584 |
|  | D max 1 cc (Gy) | 67.250 | 25.720 | 70.155 | 66.831 | 37.909 | 69.959 | 0.4846 |
|  | D mean (Gy) | 22.238 | 4.969 | 46.705 | 23.929 | 6.618 | 43.641 | 0.0692 |
| **LAD** | Volume (cc) | 3.176 | 1.112 | 5.272 | 3.176 | 1.048 | 5.272 | 0.3125 |
|  | D max (Gy) | 18.642 | 4.113 | 56.238 | 6.054 | 1.822 | 16.494 | **0.0000** |
|  | D max 0.03cc (Gy) | 18.219 | 4.030 | 54.688 | 5.989 | 1.822 | 16.100 | **0.0000** |
|  | D max 1cc (Gy) | 9.772 | 1.650 | 47.053 | 3.699 | 1.113 | 8.023 | **0.0000** |
|  | D mean (Gy) | 7.794 | 1.201 | 34.257 | 3.031 | 0.942 | 6.264 | **0.0000** |
|  | V30 Gy (%) | 0.000 | 0.000 | 63.095 | 0.000 | 0.000 | 0.000 | **0.0078** |
|  | V15 Gy (%) | 11.111 | 0.000 | 91.667 | 0.000 | 0.000 | 2.439 | **0.0000** |
| **Sinus coronaris** | Volume (cc) | 1.160 | 0.000 | 3.256 | 1.312 | 0.000 | 3.384 | 0.1250 |
|  | D max 0.03 cc (Gy) | 2.193 | 0.000 | 28.496 | 2.341 | 0.000 | 20.018 | 0.4030 |
|  | D max 1 cc (Gy) | 1.353 | 0.000 | 8.739 | 1.354 | 0.000 | 11.396 | 0.1692 |
|  | D mean (Gy) | 1.829 | 0.000 | 17.806 | 1.993 | 0.000 | 15.809 | 0.1551 |
| **Left coronary artery** | Volume (cc) | 0.288 | 0.072 | 0.952 | 0.280 | 0.072 | 0.952 | 0.4375 |
|  | D max 0.03 cc (Gy) | 25.073 | 6.580 | 53.329 | 6.744 | 3.685 | 32.018 | **0.0000** |
|  | D max 1 cc (Gy) | 0.000 | 0.000 | 0.000 | 0.000 | 0.000 | 0.000 | 1.000 |
|  | D mean (Gy) | 19.376 | 4.652 | 50.946 | 6.592 | 3.131 | 11.555 | **0.0000** |
| **Pulmonary artery** | Volume (cc) | 71.137 | 47.537 | 139.231 | 71.137 | 47.537 | 139.234 | 0.9190 |
|  | D max 0.03 cc (Gy) | 68.899 | 65.433 | 71.379 | 68.379 | 61.224 | 71.319 | 0.1399 |
|  | D max 1 cc (Gy) | 67.466 | 46.200 | 69.551 | 67.288 | 40.990 | 69.846 | 0.0552 |
|  | D mean (Gy) | 35.428 | 16.134 | 51.099 | 25.535 | 8.348 | 42.331 | **0.0000** |
| **Superior vena cava** | Volume (cc) | 5.224 | 2.024 | 13.656 | 5.224 | 2.024 | 13.744 | 1.000 |
|  | D max 0.03 cc (Gy) | 42.888 | 8.769 | 68.379 | 32.053 | 4.279 | 69.370 | 0.5460 |
|  | D max 1 cc (Gy) | 29.661 | 6.860 | 66.956 | 27.100 | 2.761 | 68.216 | 0.4270 |
|  | D mean (Gy) | 26.040 | 5.390 | 65.847 | 22.859 | 2.488 | 66.409 | **0.0410** |
| **Left atrium** | Volume (cc) | 68.832 | 54.535 | 169.360 | 68.832 | 54.535 | 169.325 | 0.0423 |
|  | D max 0.03 cc (Gy) | 67.339 | 12.599 | 70.999 | 61.578 | 6.009 | 69.919 | 0.0762 |
|  | D max 1 cc (Gy) | 51.730 | 10.077 | 68.550 | 53.410 | 5.083 | 68.409 | 0.0619 |
|  | D mean (Gy) | 14.617 | 2.178 | 40.115 | 11.559 | 2.216 | 28.905 | **0.0013** |
| **Right atrium** | Volume (cc) | 73.016 | 44.010 | 123.112 | 77.504 | 47.696 | 123.440 | 0.3257 |
|  | D max 0.03 cc (Gy) | 19.099 | 1.609 | 71.119 | 13.999 | 1.729 | 68.979 | **0.0123** |
|  | D max 1 cc (Gy) | 10.448 | 1.458 | 68.305 | 12.294 | 1.570 | 67.736 | **0.0229** |
|  | D mean (Gy) | 4.020 | 0.807 | 33.310 | 3.841 | 0.843 | 27.352 | **0.0076** |
| **Left ventricle** | Volume (cc) | 188.456 | 73.953 | 282.952 | 189.104 | 126.480 | 282.947 | 0.8358 |
|  | D max 0.03 cc (Gy) | 13.169 | 2.619 | 70.509 | 7.283 | 1.719 | 69.899 | **0.0001** |
|  | D max 1 cc (Gy) | 9.530 | 2.353 | 68.875 | 5.632 | 1.450 | 66.470 | **0.0000** |
|  | D mean (Gy) | 2.649 | 0.846 | 25.066 | 1.918 | 0.631 | 9.143 | **0.0000** |
|  | V5Gy (%) | 9.547 | 0.000 | 99.984 | 0.938 | 0.000 | 72.920 | **0.0000** |
|  | V23 Gy (%) | 0.000 | 0.000 | 45.679 | 0.000 | 0.000 | 6.565 | 0.0020 |
| **Left ventricel wall** | Volume (cc) | 90.544 | 33.723 | 251.033 | 90.816 | 62.808 | 251.032 | 0.5549 |
|  | D max 0.03 cc (Gy) | 13.169 | 2.529 | 71.199 | 7.019 | 1.649 | 71.389 | **0.0001** |
|  | D max 1 cc (Gy) | 9.295 | 2.240 | 70.004 | 4.783 | 1.300 | 70.694 | **0.0000** |
|  | D mean (Gy) | 2.948 | 0.790 | 55.548 | 1.813 | 0.580 | 53.737 | **0.0000** |
| **Right ventricle** | Volume (cc) | 106.496 | 38.070 | 151.504 | 106.496 | 62.592 | 152.264 | 0.3134 |
|  | D max 0.03 cc (Gy) | 13.579 | 1.863 | 44.259 | 6.649 | 1.716 | 15.319 | **0.0001** |
|  | D max 1 cc (Gy) | 11.150 | 1.653 | 37.038 | 4.869 | 1.365 | 11.463 | **0.0000** |
|  | D mean (Gy) | 2.575 | 0.620 | 13.445 | 1.530 | 0.468 | 4.790 | **0.0000** |
| **Lungs** | Volume (cc) | 3478.140 | 2066.094 | 5670.992 | 3478.140 | 2390.985 | 5669.096 | 0.6648 |
|  | MLD (Gy) | 13.523 | 5.067 | 21.254 | 13.948 | 5.108 | 21.985 | **0.0000** |
|  | V5 Gy (%) | 61.922 | 25.759 | 90.333 | 56.284 | 26.216 | 87.351 | 0.6790 |
|  | V20 Gy (%) | 21.675 | 6.508 | 36.252 | 25.529 | 6.581 | 38.368 | **0.0000** |
|  | V30 Gy (%) | 11.639 | 2.542 | 28.380 | 16.046 | 2.441 | 27.239 | **0.0000** |
| **Ipsilateral lung** | Volume (cc) | 1661.910 | 757.512 | 3032.751 | 1661.907 | 778.295 | 3032.692 | 0.3701 |
|  | D mean (Gy) | 19.118 | 6.319 | 33.500 | 20.863 | 5.994 | 35.780 | **0.0001** |
| **Contralateral lung** | Volume (cc) | 1738.890 | 959.488 | 3450.611 | 1756.170 | 959.488 | 3450.544 | 0.6923 |
|  | D mean (Gy) | 7.292 | 1.907 | 12.400 | 8.222 | 3.021 | 12.574 | **0.0521** |
| **Esophagus** | Volume (cc) | 36.720 | 19.737 | 61.904 | 36.720 | 24.813 | 61.904 | 0.8982 |
|  | D mean (Gy) | 17.951 | 2.713 | 33.283 | 18.908 | 4.226 | 30.202 | 0.0121 |
|  | D max (Gy) | 66.327 | 23.511 | 70.914 | 65.996 | 25.809 | 70.061 | 0.8408 |
|  | V55 Gy (%) | 7.570 | 0.000 | 41.040 | 12.386 | 0.000 | 35.282 | **0.0317** |
|  | V60 Gy (%) | 3.314 | 0.000 | 35.841 | 3.479 | 0.000 | 31.211 | 0.2063 |
|  | V60 Gy (cc) | 1.013 | 0.000 | 7.744 | 1.264 | 0.000 | 8.073 | 0.0655 |
|  | Volume included in 105% isodose (cc) of prescribed dose | 0.000 | 0.000 | 0.277 | 0.000 | 0.000 | 0.103 | 0.1250 |

PTV: planning target volume; GTV: gross tumor volume. SD. standard deviation. LAD: left anterior discending coronary artery; D mean: Mean dose; D max: maximal dose; MLD: mean lung dose; cc: cubic centimeter.
